# Identifying Social Need Trends Among Emergency Department Super-Users: A Latent Class Analysis

**DOI:** 10.5811/westjem.58307

**Published:** 2026-07-28

**Authors:** Shwath Kumaravel, Emily Parker, Eleanor Friedman, Kimberly A. Stanford

**Affiliations:** * University of Chicago, Department of Emergency Medicine, Chicago, Illinois; † University of Chicago, Section of Infectious Diseases and Global Health, Department of Medicine, Chicago, Illinois

## Abstract

**Introduction:**

Frequent emergency department (ED) users, particularly those with high rates of use, often experience complex social determinants of health (SDOH) that contribute to their repeated visits and may lead to adverse health outcomes. Our objective in this study was to characterize the needs most commonly affecting this population, which may support the creation of tailored interventions with a higher chance of success.

**Methods:**

This was a retrospective analysis of patients evaluated by an ED social medicine team in a large urban academic ED from May 2021–December 2023. Patients were identified either via frequent ED use (patients with the 10 highest number of visits each month) or referred by ED staff. We assigned 11 categories of medical, mental health, and social needs through chart review and team documentation. The primary outcome was the identification of distinct need-based subgroups among patients with high ED use, using latent class analysis of documented social, mental health, and medical needs.

**Results:**

Of the 181 patients, representing approximately 0.4% of the ED patient population, 149 (82.3%) were frequent ED users, with a median of 14 ED visits annually (IQR 8–21), while the rest were referred by ED staff (median 2 visits (1–4.25). The majority were non-Hispanic Black (159, 87.8%) and publicly insured (163, 90.0%). The most common needs were related to housing instability (116, 64.1%) and limited access to outpatient care (112, 61.9%). Latent class analysis revealed four distinct subgroups, all of which included limited access to outpatient care: 1) primarily limited access to outpatient care (40, 22.1%); 2) housing instability and other basic needs (27, 14.9%); 3) housing instability, psychotic disorders, other basic needs, and inability to live independently (23, 12.7%); and 4) housing instability, substance use, and psychotic disorders (91, 50.3%).

**Conclusion:**

This analysis identified four distinct need-based subgroups of frequent ED users, suggesting the need for tailored, multifaceted interventions. Housing instability, psychiatric illness, substance use, and outpatient care access were prevalent across groups, underscoring the importance of including resources for these basic needs in any programs developed for this population. Findings support prioritizing coordinated care models, particularly those that incorporate housing, behavioral health, substance use resources, and navigation support, for this vulnerable population.

## INTRODUCTION

Social determinants of health (SDOH) have been shown to negatively impact the health outcomes of individuals.^1^,^2^ Defined by the World Health Organization as the “non-medical factors [such as income, education, employment status, housing, and access to proper care] that influence health outcomes,”^3^ it is estimated that more than half of the U.S. population is affected by at least one adverse SDOH.^4^,^5^ For example, around 26.4 million American households are uninsured,^6^,^7^ which prevents them from being able to seek the routine care they need, resulting in worse health outcomes,^8^,^9^ higher rates of chronic diseases,^5^,^10^–^12^ and increased reliance on the emergency department (ED) for care.^13^

Emergency departments play a unique role in the healthcare system because, in addition to treating emergent conditions, they serve as a public health safety net, an entry point into the medical system, and sometimes the sole source of medical care and social services for individuals with limited access to care. Frequent ED users, commonly defined as individuals who present to the ED more than four to six times in a given year,^14^ have been found to have a disproportionate burden of SDOH.^14^,^15^ It is estimated that frequent ED users account for 3.816% of all ED users but 14–47% of all ED visits.^11^ Prior studies have found that frequent ED users are more often publicly insured, have a disproportionate burden of chronic diseases and unmet primary care needs, perceive their own health status as poor, and have a higher likelihood of substance abuse, unemployment, mental health issues, and unstable housing.^11^,^16^

While a growing body of literature has examined frequent ED users, the population is somewhat ambiguously defined and generally includes individuals with visit rates more than four per year.^11^,^13^,^17^,^18^ However, given the definition’s inconsistency as well as the population’s unique characteristics, the subset of this population with the highest reliance on the ED for care tends to be underexamined.^19^ These “super-utilizers”^18^ are sometimes defined as those who visit the ED > 18 times in a year. However, while there are likely important differences between individuals who visit the ED four times a year and those who visit three or four times that number, a standard definition of the minimum visit rate for this group has not been established. A few studies have suggested that the individuals with the highest number of ED visits are more likely to be uninsured, have lower acuity visits,20,21 and tend to visit the ED in place of other healthcare sources.^18^ Despite their disproportionate number of ED visits, little is known about these super-users, how different their needs may be from patients with a lower ED use rate, and what types of interventions would be most beneficial for them.

In 2021, a large urban academic hospital ED on the South Side of Chicago, an area of high economic hardship, implemented an ED social medicine team as a quality improvement (QI) initiative, modeled after a similar program in San Francisco.^22^ The team included social workers, care coordinators, patient navigators, behavioral health specialists, pharmacists, and an emergency physician. Team members met monthly to discuss patient cases and coordinate interventions. This multidisciplinary team model allowed for more comprehensive interventions for individuals with greater needs, as identified by high visit rates or clinician assessment, and the monthly meetings provided an opportunity for in-depth review of charts and group discussion of all potentially relevant resources. This project aims to review this cohort of individuals, most of whom would be considered not just frequent ED users, but super-users, with ED visit rates well above four per year, to understand the drivers of their ED use, with the ultimate goal of developing more efficient and effective interventions to better meet the specific needs of the most frequent ED users.

**Population Health Research Capsule**

What do we already know about this issue?

*Frequent emergency department (ED) users report substantial social and medical needs, but the highest users are poorly characterized and lack targeted interventions*.

What was the research question?

*What distinct need-based subgroups exist among frequent ED users?*

What was the major finding of the study?

*Latent class analysis identified four subgroups; housing instability (64%) and limited access to outpatient care (62%) were the most common needs*.

How does this improve population health?

*Identifying major needs and subgroups enables development of targeted, coordinated interventions to reduce ED use among the highest users*.

## METHODS

We conducted a retrospective review of all individuals followed by the social medicine team from its inception in May 2021 through December 2023. Patients were identified for team intervention either through frequent ED visits (among the top 10 most frequent ED users in the calendar month prior to the team meeting) or referral from ED staff. We performed case selection criteria, variable definition, abstraction forms, missing-data management planning, abstractor training, performance monitoring, and medical record identification in line with recommended medical record review criteria.^23^ We recorded interventions by the ED social medicine team for clinical care in a REDCap secure database,^24^,^25^ which was supplemented with manual chart review. Demographic information, reasons for individual ED visits, and SDOH were extracted for each patient.

For each individual, it was noted whether they had been referred to the team by ED staff or identified via the monthly frequent ED users report. And for each of them we recorded the number of ED visits at the primary project site during the 12 months after that individual’s initial evaluation by the ED social medicine team. Although some visits to other EDs are visible in the electronic health record (EHR), many local EDs do not use the same EHR system and, thus, visits outside the primary site ED could not be reliably tracked. Because of this uncertainty, only visits at the primary site were included. Of note, patients with ED visits solely due to unavoidable medical conditions and those visiting primarily for sickle cell pain crises were not eligible for the ED social medicine team intervention, because these visits were considered medically unavoidable and not related to SDOH.

### Categorization of Patient Needs

We created 11 categories of patient needs (Table 1) based on our clinical experience, frequently occurring themes in the database, and review of the literature. Categories were assigned to each individual based on group discussion by the ED social medicine team, as documented in the database, as well as review of the medical chart by two independent reviewers. Individuals were assigned to a category if it was noted during at least one ED visit, and individuals could be assigned to multiple categories.

### Statistical Analysis

We characterized individuals in the social medicine team’s database descriptively based on their sociodemographic information and their reported needs. We used medians and interquartile ranges (IQR) for continuous variables (such as visit rates) and frequencies and percentages to describe the population for categorical variables. We used a chi-square test of independence to determine statistical significance between groups.

Latent class analysis was used to determine groupings of needs in the patient population. We used poLCA v. 1.6.0.1,^26^ a latent class analysis package built for R (R Foundation for Statistical Computing, Vienna, Austria),^27^ and the model was run with 2–10 clusters. The optimal number of clusters was determined by the lowest value of the Akaike information criterion.^28^–^30^ The model was validated by observing lower off-diagonal values and higher diagonal values in the posterior probability matrix.^29^ We ran the poLCA model 1,000 times with five different random starting positions to account for cluster assignment variation among study subjects. Cluster assignment was determined by the frequency of cluster assignment. Model fit and validity were assessed using Akaike information criterion and Bayesian information criterion for class selection, maximum likelihood estimation with multiple random starting values to ensure convergence and avoid local maxima, and posterior class membership probabilities to evaluate classification certainty. All data analysis was performed using R software v4.0.3.

This project was formally determined to be QI, not human subjects research; therefore, it was not overseen by the institutional review board, per institutional policy.

## RESULTS

The ED social medicine team evaluated 181 patients from May 2021–December 2023. Of these, 149 (82.3%) were frequent ED users, identified via the monthly ED use report, and 32 (17.7%) were referred by ED staff. Frequent ED users had a median of 14 visits (IQR 8–21) to the primary project ED in the initial 12 months after evaluation by the social medicine team. Many of these individuals had visits to other EDs during this period as well, but these could not be reliably quantified from the EHR and were not included. Among individuals referred by staff, the median was two visits (IQR 1, 4.25) to the primary project ED, with a maximum of 16. Overall, 51 (28.2%) of the population had more than 18 visits in 12 months.

Among all patients, the median age was 55 (IQR 36–65), and 58% were male (Table 2). Most of the patients identified by the ED social medicine team were non-Hispanic Black (87.8%), reflecting the racial demographics of the local ED population, and used public insurance (92.8%), with 6.1% uninsured. The distribution of demographics was similar between the two groups, except that frequent ED users notably had a higher percentage of males than females (61.7% vs 40.6%) compared to referred patients. At the end of the evaluation period, 13 (7.18%) individuals were confirmed deceased, 11 of whom were frequent ED users.

Table 3 shows the frequency of each recorded need in the population. The two most common needs observed in the population were housing instability (116, 64.1%) and limited access to outpatient care (112, 61.9%). Overall, 64 (35.4%) had a history of substance use disorder, 76 (42%) had difficulty meeting basic needs, and 64 (35.4%) had a history of a psychotic disorder.

Some differences were observed between frequent ED users and the individuals referred to the ED social medicine team, although most of these were not statistically significant. Frequent ED users were more frequently noted to have substance use disorder (37.6% vs 25.1%), psychiatric illness (psychotic disorder 38.3% versus 21.9%, anxiety/depression 23.5% versus 15.6%), and other basic needs such as lack of a cellphones or reliable transportation (43.6% versus 34.4%), while referred patients more frequently were unable to live independently (28.9% versus 43.8%).

We performed latent class analysis for the entire ED social medicine team population and identified four distinct subgroups characterized by the predominant needs of each group (Table 4), all of which included limited access to outpatient care: 1) primarily needed assistance accessing outpatient care; 2) housing instability and other basic needs; 3) housing instability, other basic needs, inability to live independently, and psychotic disorders; and 4) housing instability, substance use, and psychotic disorders. Subgroup 4 was the largest group, with 50.3% in this subgroup. Access to outpatient hemodialysis was noted in subgroup 1 (23%) and subgroup 4 (4%). Housing instability was highly prevalent across all subgroups, ranging from 40% in subgroup 1 to 96% in subgroup 2. The median number of visits in 12 months after involvement of the ED social medicine team was similar between the subgroups (14, 12, 12, 10, respectively), with a relatively high IQR (6–19.3, 6.5–20, 2.5–19.5, 6–19) in all subgroups, reflecting the presence of very high users in each subgroup. Frequent ED users in subgroup 4 had a median visit number of 12.5 (IQR 13 : [8,21]). Referred individuals comprised a larger proportion of subgroups 1 and 3 (22.5% and 26.1%, respectively) compared to subgroups 2 and 4 (7.41% and 16.5%, respectively).

## DISCUSSION

To date, the literature has not focused on frequent ED users with extremely high ED visit rates. In this sample, frequent ED users had a median 12-month ED visit rate well above the threshold of four visits per year that is commonly used to define frequent ED use, and more than a quarter had over 18 visits per year. In one study of Medicaid data, the medical needs and SDOH of very high-use ED patients correlated significantly with those noted in the largest subgroup in this project, suggesting these may be common needs that will be key to address in future interventions. The combination of substance abuse, housing needs, and mental illness, which were major drivers identified in the largest subgroup in this study, has been shown to increase the likelihood of ED use in other studies as well.^31^–^34^ “Housing first” programs may decrease hospital and ED use, even without integrated substance use interventions,^35^,^36^ but they could be even more effective when using a holistic approach that includes substance use and mental health resources.^37^ These interventions can be implemented directly from the ED visit,^38^ which may be a critical opportunity for engagement in a housing-first model.

Interestingly, the group of individuals defined by unstable housing, substance use, and mental health needs, which constituted the largest subgroup, had a lower median ED visit rate than frequent ED users overall. While we were unable to examine resource use outside the primary site ED, members of this group were frequently documented to visit multiple EDs around the city, spend prolonged periods incarcerated, or occasionally be admitted to psychiatric hospitals. This may account for sporadic heavy use of the primary site ED, followed by periods of absence, creating the illusion of lower ED use when, in fact, this group’s overall use of the ED and system resource may have been much higher—an important area to consider in future research.

As has been reported in previous studies,^11^,^14^,^39^–^41^ most individuals in this QI project used either Medicaid or Medicare. Evidence suggests that Medicaid users experience disproportionate difficulty accessing care, including being less likely to have a usual source of care, facing long wait times and low appointment availability, and transportation issues,^42^–^44^ which is associated with higher ED use.^44^,^45^ While some studies have suggested that individuals with the highest ED use may not represent a burden on ED costs or crowding due to their relatively low prevalence and low-acuity visits,^20^ in this QI project some individuals were visiting the ED almost daily for a period of weeks to months. Further study is needed to understand the impact of this type of heavy ED use, in particular given low Medicaid reimbursement rates and bed use by uninsured and underinsured patients.

This analysis identified four major subgroups, or clinical phenotypes, of individuals who were evaluated by the ED social medicine team. While some characteristics, such as housing instability, psychiatric illness, and access to outpatient care, were present in all subgroups, differences between the subgroups suggest that tailored interventions could be developed to target the specific needs of each subgroup more efficiently. For instance, subgroup 4, the largest subgroup, experienced the confluence of mental health, housing instability, and substance use, suggesting that more resources should be put toward comprehensive interventions that target all these needs simultaneously, such as housing programs incorporating substance use treatment and mental health support on site. While these needs have been identified among patients with repeated hospitalizations,^46^ this study is novel in its focus specifically on the highest users of ED care, as well as the amount of data available regarding distinct SDOH. Existing literature generally focuses on those with more than four or five annual ED visits,^47^,^48^ which may not be representative of the needs of this very specific subset of individuals. Given the prevalence of housing instability, psychiatric illness, and access to outpatient care in all subgroups, all interventions for a similar population should consider including housing, mental health support, and community health workers or care navigators to assist individuals in accessing the care they need. Some existing interventions have already shown promise in improving outcomes for this group of individuals, but increased support for these programs is needed from health departments and other government agencies that could provide funding or infrastructure that individual EDs cannot develop or sustain alone. Implementation research examining service delivery strategies in a variety of hospital settings and providing evidence of feasibility and efficacy is urgently needed.

## LIMITATIONS

This was a single-center QI initiative and, as such, results may not be generalizable to other locations. However, the findings are consistent with existing literature, and they are likely applicable to other urban EDs serving socioeconomically depressed populations. Additionally, because the establishment of an ED social medicine team was a QI initiative, team structure, record keeping, and interventions evolved over time. Identification of social needs is somewhat subjective, and this review relied on needs being recorded in a database or in the medical record, which may not always have been consistently done.

Individuals may not have disclosed certain SDOH to their medical team, and if not relevant to their ED visit, these needs may not have been recorded in the chart. Some individuals had more comprehensive social work evaluations, while others were unable or unwilling to participate in these evaluations, which may have affected the team’s ability to accurately identify their SDOH. Individuals identified to have only medical needs driving their ED visits, including sickle cell pain crises, were excluded, but it is possible that they, too, had unidentified SDOH. Lastly, because members of the ED social medicine team were not reliably able to access information about ED visits or medical admissions at other hospitals, psychiatric admissions, or incarceration, use of system resources could not be completely evaluated. However, the patterns observed in this QI project are likely similar to those of ED super-users elsewhere, and the findings about ED use at a single site are relevant to other EDs caring for similar populations.

## CONCLUSION

Frequent users of the emergency department contribute to crowding and economic consequences; at the same time, they have worse health outcomes than other ED visitors. Needs identified in this analysis—most notably housing instability, psychiatric illness, substance use, and difficulty accessing outpatient care—could be used to develop targeted, comprehensive interventions that might have a higher likelihood of success than the current piecemeal system employed in most hospitals. As social safety nets face increasing challenges, placing additional strain on EDs and social service workers, it will be critical to advocate for additional, evidence-based resources that effectively address the most pressing needs of this vulnerable group.

## Figures and Tables

**Table 1:** Major categories of social, medical and mental health needs of emergency department frequent users as determined through literature review, common themes in the data, and author experience.

Need category	Definition
Social needs	
Housing instability	Currently homeless or at risk of homelessness
Other basic needs	Limited access to other basic needs (eg, food, a cellphone, insurance)
Financial instability	Difficulty paying bills or reports financial strain
Access to outpatient care	Difficulty making or attending routine appointments
Interpersonal violence	Physical or sexual abuse from an intimate partner or other individual
Mental health needs	
Anxiety/depression	Depression or anxiety as one of the primary motivators for at least one ED visit
Psychotic disorders	Schizophrenia or other psychotic disorders contributing to or with symptoms present during at least one ED visit
Substance use disorder	Abuse of or dependence on alcohol, marijuana or illicit substances; or ED visits primarily seeking narcotics
Medical needs	
Serious medical conditions	ED visit primarily due to a serious medical condition unable to be managed outpatient
Access to hemodialysis	ED visit due to lack of access to regular hemodialysis
Unable to live independently	Requires additional assistance at home (eg, home health care) or placement (e.g., skilled nursing facility)

*ED*, emergency department.

**Table 2:** Demographics of patients evaluated by the emergency department (ED) social medicine team from May 2021 to December 2023 by source, referred by ED staff vs frequent ED users.

Variable	Total, N (%)	Referred, n(%)	Frequent emergency department users, n(%)
Total	181 (100)	32 (17.7)	149 (82.3)
Gender			
Male	105 (58.0)	13 (40.6)	92 (61.7)
Female	72 (39.8)	18 (56.3)	54 (36.2)
Transgender Female	1 (0.6)	0 (0)	1 (0.7)
Transgender Male	0 (0)	0 (0)	0 (0)
Other	3 (1.7)	1 (3.1)	2 (1.3)
Age			
18–39	59 (32.6)	11 (34.4)	48 (32.2)
40–59	55 (30.4)	5 (15.6)	50 (33.6)
60–79	58 (32.0)	13 (40.6)	45 (30.2)
≥ 80	9 (5.0)	3 (9.4)	6 (4.0)
Race/Ethnicity			
Hispanic Black	2 (1.1)	0 (0)	2 (1.3)
Hispanic White	2 (1.1)	1 (3.1)	1 (0.7)
Non-Hispanic Black	159 (87.8)	29 (90.6)	130 (87.2)
Non-Hispanic White	8 (4.4)	1 (3.1)	7 (4.7)
Hispanic/Latino	2 (1.1)	0 (0)	2 (1.3)
Other/Unknown	8 (4.4)	1 (3.1)	7 (4.7)
Deceased during project	13 (7.2)	2 (6.3)	11 (7.4)
Insurance coverage			
Medicare	23 (12.7)	4 (12.5)	19 (12.8)
Medicaid	111 (61.3)	16 (50)	95 (63.8)
Medicare + Medicaid	29 (16)	6 (18.8)	23 (15.4)
Private	5 (2.8)	2 (6.3)	3 (2.0)
Other	2 (1.1)	0 (0)	2 (1.3)
Uninsured	11 (6.1)	4 (12.5)	7 (4.7)

**Table 3:** Reported medical and social needs of individuals evaluated by the emergency department (ED) social medicine team from May 2021–December 2023 by source, referred by ED staff vs frequent ED users.

Needs	Total N (%)	Referred n(%)	Frequent emergency department users n (%)	*P* - value
	181 (100)	32 (17.7)	149 (82.3)	
Social Needs				
Housing instability	116 (64.1)	20 (62.5)	96 (64.4)	.84
Other basic needs	76 (42.0)	11 (34.4)	65 (43.6)	.34
Financial instability	38 (21.0)	11 (34.4)	27 (18.1)	.04*
Access to outpatient care	112 (61.9)	20 (62.5)	92 (61.7)	.94
Interpersonal violence	14 (7.7)	4 (12.5)	10(6.7)	.27
Mental Health Needs				
Anxiety/Depression	40 (22.1)	5 (15.6)	35 (23.5)	.33
Psychotic disorders	64 (35.4)	7 (21.9)	57 (38.3)	.08
Substance use disorder	64 (35.4)	8 (25.0)	56 (37.6)	.18
Medical Needs				
Serious medical conditions	38 (21.0)	6 (18.8)	32 (21.5)	.73
Access to hemodialysis	13 (7.2)	1 (3.1)	12 (8.1)	.33
Unable to live independently	57 (31.5)	14 (43.8)	43 (28.9)	.10

**Table 4:** Reported social, mental health, and medical needs of individuals evaluated by the emergency department social medicine team from May 2021–December 2023 by subgroup, classified using latent class analysis.

	Total N (%)	Subgroup 1 n (%)	Subgroup 2 n (%)	Subgroup 3 n (%)	Subgroup 4 n (%)
Total	181 (100%)	40 (22.1%)	27 (14.9%)	23 (12.7%)	91 (50.3%)
Referred	32 (17.7%)	9 (22.5%)	2 (7.4%)	6 (26.1%)	15 (16.5%)
Frequent users	149 (82.3%)	31 (77.5%)	25 (92.6%)	17 (23.9%)	76 (83.5%)
Social needs					
Housing instability	116 (64.1%)	16 (40%)	26 (96.3%)	15 (65.2%)	59 (64.8%)
Other basic needs	76 (42.0%)	11 (27.5%)	16 (59.3%)	23 (100%)	26 (28.6%)
Financial instability	38 (21%)	8 (20%)	5 (18.5%)	10 (43.5%)	15 (16.5%)
Access to outpatient care	112 (61.9%)	20 (50%)	14 (51.9%)	22 (95.7%)	56 (61.5%)
Interpersonal violence	14 (7.7%)	1 (2.5%)	4 (14.8%)	4 (17.4%)	5 (5.5%)
Mental health needs					
Anxiety/depression	40 (22.1%)	8 (20%)	8 (29.6%)	4 (17.4%)	20 (22%)
Psychotic disorders	64 (35.4%)	7 (17.5%)	6 (22.2%)	12 (52.2%)	39 (42.9%)
Substance use disorder	64 (35.4%)	15 (37.5%)	4 (14.8%)	0 (0%)	45 (49.5%)
Medical needs					
Serious medical conditions	38 (21%)	7 (17.5%)	5 (18.5%)	0 (0%)	26 (28.6%)
Access to hemodialysis	13 (7.2%)	9 (22.5%)	0 (0%)	0 (0%)	4 (4.4%)
Unable to live independently	57 (31.5%)	14 (35%)	8 (29.6%)	16 (69.6%)	19 (20.9%)
